# Analysis of inflammatory and metabolic biomarkers in patients submitted to abdominoplasty after bariatric surgery^1^

**DOI:** 10.1590/s0102-865020190050000006

**Published:** 2019-06-03

**Authors:** Miguel Luiz Antônio Modolin, Wilson Cintra, Rodrigo Itocazo Rocha, Cristina Pires Camargo, Nádia de Rosso Giuliani, Heraldo Possolo de Souza, Hermes Vieira Barbeiro, Rolf Gemperli

**Affiliations:** I Assistant Physician, Plastic Surgery and Burns Division, Clinics Hospital, Medical School, Universidade de São Paulo (USP), Brazil. Interpretation of data, statistics analysis, critical revision.; II Full Professor, Plastic Surgery and Burns Division, Clinics Hospital, Medical School, USP, São Paulo-SP, Brazil. Interpretation of data, statistics analysis, critical revision.; III PhD, Plastic Surgery and Burns Division, Clinics Hospital, Medical School, USP, São Paulo-SP, Brazil. Interpretation of data, statistics analysis, critical revision.; IV Intern Physician, Internship in Specialized Complementation, Body Contour Group, Plastic Surgery and Burns Division, Clinics Hospital, Medical School, USP, São Paulo-SP, Brazil. Interpretation of data, statistics analysis, critical revision.; V Full Professor, Medical Emergencies Discipline, Medical School USP, São Paulo-SP, Brazil. Interpretation of data, statistics analysis, critical revision.; VI PhD in Sciences,Medical School, USP, São Paulo-SP, Brazil. Interpretation of data, statistics analysis, critical revision.

**Keywords:** Obesity, Metabolic Syndrome, Interleukins, Homeostasis, Abdominoplasty

## Abstract

**Purpose:**

To evaluate the serum variations of Interleukins (Il) and CPR of abdominoplasties in post-bariatric patients and, to equate the homeostasis (HOMA) from the variations of glycemia and insulin to evolute the metabolic modifications.

**Methods:**

Fourteen women were submitted to abdominoplasties with weight loss after a gastroplasty. Levels of IL_4_, IL_6_, IL_10_, CRP, glycemia and insulin were obtained during the pre-operative, trans-operative, 24 hours post, 7^th^ and 14^th^ postoperative days.

**Results:**

The IL_4_ was higher at 24 hours post-surgery, and after a moderate decrease, it remained high until the 14^th^ day. The IL_6_ and CRP had an expressive increase during the trans-operative period. The CRP remained high, and the IL_6_ decreased on the 7^th^ and 14^th^ days. The IL_10_ increased during the transoperative period, and it posteriorly decreased to lower levels in comparison to the pre-operative period. The already increased glycemia during the pre-operative period was even higher during the trans-operative and then, returned to preliminary values on the 7^th^ and 14^th^ days after surgery. The HOMA accompanied the insulin.

**Conclusion:**

The inflammatory and glycemic serum levels decrease after abdominiplasty in obese post-bariatric patients.

## Introduction

Different studies prove that the adipose tissue - subcutaneous and visceral - has endocrine activity producing a conjunct of proteins, generically denominated as adipokines. From these adipokines, the cytokines are noted due to inflammatory and anti-inflammatory activities known as interleukins (IL)^1^ ^,^ ^2^.

These interleukins have their levels increased in obesity, and they are responsible for a chronic inflammatory state stimulating the hepatic production of the C-reactive protein (CRP)^3^ ^-^ ^5^. Together with the IL6, the CRP promotes an increase of the peripheral resistance to insulin with an eventual diabetes case in predisposing obese patients^6^ ^-^ ^8^. Hence the reason why obesity is considered an inflammatory and metabolic disease.

Patients with a considerable weight loss and reduction of adiposity, through diet or gastroplasty, have noted changes in inflammatory markers, as the reduction of glycemic levels and decrease of the peripheral insulin resistance^9^ ^-^ ^11^. Not with standing, few patients with remaining obesity levels present inflammatory activities incipient with variable levels of inflammatory markers^12^, changes of the glycemia and insulin rates and, resulting changes in homeostasis (HOMA)^5^ ^,^ ^6^ ^,^ ^8^. 

Such patients require the removal of remaining accumulated fat in the dermis resulting from the weight loss, and the abdominoplasty is the most required surgery. In this prospective study, the variations of IL_5_, CRP, glycemia, insulin, and HOMA are described in moderately obese patients submitted to abdominoplasty in anchor at pre-preoperative, trans-operative and different days of the post-operative and possible modifications in metabolic and inflammatory syndromes. Also, to compare them with studies of other authors.

## Methods

All of them signed the informed consent to participate in the study approved by the Ethics Committee of the Clinics Hospital of the Medical School, Universidade de São Paulo (USP), registration number 48112015.0000.0068. This study was conducted at the Clinics Hospital, Medical School USP without sponsors, therefore, without any conflict of interests.

Fourteen female patients were recruited, all of them were submitted to a malabsorptive-restrictive bariatric surgery by the Capela-Fobi technique, and their demographic composes Table 1. 

**Table 1:** Demographic data of 14 female patients.

Before Gastroplasty	Before Abdominoplasty
Age	
(years) Mean - 42.35	45.92
Minimum - 21	29
Maximum - 58	60
	
BMI	
(Kg/m^2^ ) Mean - 45.63	29.62
Minimum - 35.6	21.3
Maximum - 56.7	36.7

All the patients were healthy. They were non-smokers, not under hormone therapy, reposition or contraceptive; they did not use drugs that could alter their behavior. The tests and psychological analyses demonstrated a good psycho-mental balance. 

### Surgery

 The abdominoplasty was conducted using an incision with an anchor shape, that is, one longitudinal axis delimitated by a bi-digital clamping since the xiphoid appendix that is found with an inferior cross-sectional fuse containing the lower abdominal fold and extending until the bilaterally superior iliac spines. With the bi-digital clamping maneuver, the superior resection line of this axis is delimitated. After the fat contents of these axes are removed, the musculoaponeurotic plication of the anterior abdominal wall is performed. After the synthesis of the skin and subcutaneous, in three axes, the umbilicus is externalized and fixated in the abdominal skin. The average weight of the exceeding resected adiposity was of 2.068kg, ranging between 1.000 kg and 3.600 kg.

### Biochemical analyses

 Blood samples were collected in the pre-operative (A), trans-operative (B), and 24 h after the surgery (C), in the 7^th^ day of the postoperative (D), and 14^th^ of the postoperative period (E). In these samples, IL_4_, IL_6_, IL_10_, CRP, glycemia, and insulin were measured. 

The interleukins were analyzed through the immunoassay with Miclipex magnetic beads and MagPix System (Merck Milli Bore - USA), and the results were measured in peak values grams/dl.

The C-Reactive Protein G assessed the CRP; through quantitative in vitro immunoturbidimetric CRP assay using the systems Roche/Hitachi cobas c. The values were expressed as mg/dl.

Glucose was dosed by the Glucose HK Gen.3, in vitro test using the system Roche/Hitachi cobas c. The values were expressed as mg/dl. 

The insulin was analyzed by the electrochemiluminescence immunoassay method (ECLIA) in the analyzer Roche/Hitachi cobas c. The values were expressed as mg/dl. 

The homeostasis was measured following the HOMA equation = Insulin x Glycemia/22.5. 

The parameters were assessed in mean absolute values with a mean of the standard error presented in Table 2.

**Table 2:** Results expressed as mean absolute values of the measured parameters and their respective standard errors.

	Pre (mean ± standard error mean)	Trans (mean ± standard error mean)	24hPOST (mean ± standard error mean)	7dPOST (mean ± standard error mean)	14dPOST (mean ± standard error mean)
IL-4	0.22 ± 0 .4	0.17 ± 0.01	0.37 ± 1.0	0.68 ± 1.2	0.79 ± 0.1
IL-6	3.66 ± 13.0	23.5 ± 1.7	40.43 ± 0.07	11.2 ± 8.4	4.53 ± 0.6
IL-10	6.02 ± 6.2	4.6	11.48 ± 0.5	6.18 ± 6.3	4.59 ± 4.2
CRP	3.3 ± 7.7	1.15 ± 1.3	47.9 ± 1.7	27.85 ± 19.8	17.2 ± 2.7
Glycemia	99.1 ± 29.1	125.8 ± 3.8	127.6 ± 3.9	105.1 ± 36.1	101 ± 1.6
Insulin	4.1 ± 3.4	4.25 ± 0.2	15.8 ± 1.4	10.2 ± 12.4	8.4 ± 0.6
HOMA	18.05	23.5	89.6	47.6	38.2

## Results

The IL_4_ levels increased since the first 24 h after surgery, and they remained high until the 14^th^ postoperative day. The IL_6_ measures increased in the trans- and 24 h postoperative, and then dropped in the 7^th^ postoperative day approximating to the initial level on the 14^th^ postoperative day; these variations are similar to the IL_10_ with excretion in the 24h postoperative when it presented a decrease. The CRP was considerably higher at 24 hours after surgery and decreased on days 7^th^ and 14^th^ post-surgery.

Glycemia increased in the trans-operative and the 24 h post-surgery, and decrease to normal levels on the 7^th^ and 14^th^ postoperative days. The insulin level behavior followed the linear pattern of the glycemic levels. The relationship between glycemia and insulin presented by HOMA was higher in the trans- and 24 h postoperative moments, although it continued high in the 7^th^ and 14^th^ postoperative days when compared to the pre-operative phase. 

## Discussion

The adipose tissue performs an endocrine activity, producing several proteins as such IL and adipokeins. These proteins play an crucial function in obesity related complications. Most of these complications were associated to paracrine inflammation adipose tissue’ role. The inflammation causes metabolic dysfunstions and type 2 diabetes^2^.

In this study, the aim was to identify the modifications in the interleukins IL_4,_ IL_6,_ and IL_10._ The IL_4_ is an interleukin produced in the adipose tissue as a result of the interaction with macrophages residing in this tissue and others that flow to it also with the inflammatory exudate after the surgical trauma^11^ ^,^ ^12^. In the visceral adipose tissue, the IL_4_ regulates the lipogenesis due to its lipolytic activity. In the subcutaneous adipose tissue, it performs an accentuated anti-inflammatory activity and its variations in levels are associated with the aggression of the abdominoplasty^1^ ^,^ ^3^ ^,^ ^8^. Due to its capacity to control lipogenesis and lipolysis, it collaborates to variations in the levels of fatty acids and consequently the deposit of glucose and, as a consequence, the regulatory activity of the insulin sensitivity and variations subsequent of homeostasis^13^. The patients submitted to abdominoplasty had an average BMI of 29.62 Kg/m^2^; therefore, still obese, with IL_6_ and CRP moderately higher translating a moderate inflammatory state. In the trans-operative period after the resection of the excess abdominal fat, there was a significant increase of the IL_6_; in the immediate post-operative there was a high increase of the IL_6_ and CRP sending the neuroendocrine stimuli corresponding to the inflammatory response to trauma. In sequence, on postoperative days 7^th^ and 14^th^, there was a reduction of the IL_6_ levels accompanied by the reduction of CRP levels, although still high, they proved the continuation of the inflammatory state^9^, considering that in this series, even with the removal of a mean adipose mass of 1.92kg and reducing the mean BMI to 28.76 kg/m^2^ corresponding to a medium level of obesity with consonant inflammatory state. 

Modifications in the IL_10_ levels were found in the results. This cytokine is released due to stimuli exercised over the monocyte/macrophages located in the adipose tissue, and it has anti-inflammatory action^1^. Its profile showed an increase in the pre-operative, as in the studies series, the patients were obese with an evolving inflammatory state. The IL_10_ level was accentuated in the trans-operative period, in agreement with the consulted literature^1^ ^,^ ^10^ and as an inference, it can be hypothesized that it had an anti-inflammatory protective biological activity antagonistic to IL_6_. 

Obesity is also a metabolic disease with increases in the glycemic levels and incipient peripheral insulin resistance^14^. This scenario was seen in the studied series since the preoperative and in the trans-operative as a result of the neuroendocrine stimuli with elevated HOMA rates, meaning an increase of the peripheral insulin resistance. It is compatible with the inflammatory state caused by the incipient IL_6_ activity corroborated by the constancy of the elevated CRP levels, despite the increments of IL_4_ levels, and moderate IL_10_ levels that are anti-inflammatories^15^. 

The analyzed series in the occasion of the abdominoplasty had a mean BMI compared to the moderate level of obesity and the inflammatory markers IL_6_ and CRP showed a low inflammatory level, and the postoperative modifications demonstrated an increase of these markers, but overall, of the IL_4_ andIL_10_ anti-inflammatories, also noting that the HOMA still was elevated. However, it should be considered that although operated, the mean BMI of this series continued compatible with the moderate level of obesity. These findings are congruent with findings of other authors^1^ ^,^ ^15^, although they conducted longer studies. 

The limitations of this study were a short-term study, in this first project we only describe the data. However, in the future studies, our group will compare the obese population to a normal weight population submitted to an abdominoplasty procedure.

There for, our results when compared to the literature data^1^ ^,^ ^8^ ^,^ ^10^ ^,^ ^11^ ^,^ ^15^, showed an improvement in the metabolic syndrome in obese patients submitted to abdominoplasty in anchor.

## Conclusion

This study suggested an improvement in the metabolic syndrome in obese patients submitted to abdominoplasty in anchor.
